# A Case of Diastolic Murmur at the Base of the Heart of Anemic Origin

**Published:** 1913-10

**Authors:** 


A Case of Diastolic Murmur at the Base of the Heart of Anemic Origin, Bouchut and Mazel, *Arch. des Mal. du Coeur, des Vaisseaux, et du Sang,* 1913, VI, 191. The authors report the care of a man aged 62 years, who was suffering from chronic nephritis, hypertrophy of the heart, arterial hypertension (280 mm.) albuminuric retinitis and albuminuria, who had repeated large hemorrhages from the intestines, followed by the development of a systolic and a diastolic murmur at the base of the heart and galop rhythm.

